# The uncanny valley effect in typically developing children and its absence in children with autism spectrum disorders

**DOI:** 10.1371/journal.pone.0206343

**Published:** 2018-11-01

**Authors:** Shuyuan Feng, Xueqin Wang, Qiandong Wang, Jing Fang, Yaxue Wu, Li Yi, Kunlin Wei

**Affiliations:** 1 School of Psychological and Cognitive Sciences and Beijing Key Laboratory of Behavior and Mental Health, Peking University, Beijing, China; 2 Department of Statistical Science, School of Mathematics and Computational Science, Sun Yat-sen University, Guangzhou, Guangdong, China; 3 Southern China Research Center of Statistical Science, Sun Yat-sen University, Guangzhou, Guangdong, China; 4 Academy for Advanced Interdisciplinary Studies, Peking University, Beijing, China; 5 Peking-Tsinghua Center for Life Sciences, Academy for Advanced Interdisciplinary Studies, Peking University, Beijing, China; 6 Qingdao Autism Research Institute, Qingdao, Shandong, China; Newcastle University Institute for Health and Society, UNITED KINGDOM

## Abstract

Robots and virtual reality are gaining popularity in the intervention of children with autism spectrum disorder (ASD). To shed light on children’s attitudes towards robots and characters in virtual reality, this study aims to examine whether children with ASD show the uncanny valley effect. We varied the realism of facial appearance by morphing a cartoon face into a human face, and induced perceptual mismatch by enlarging the eyes, which has previously been shown as an effective method to induce the uncanny valley effect in adults. Children with ASD and typically developing (TD) children participated in a two-alternative forced choice task that asked them to choose one they liked more from the two images presented on the screen. We found that TD children showed the effect, i.e., the enlargement of eye size and the approaching realism reduced their preference. In contrast, children with ASD did not show the uncanny valley effect. Our findings in TD children help resolve the controversy in the literature about the existence of the uncanny valley effect among young children. Meanwhile, the absence of the uncanny valley effect in children with ASD might be attributed to their reduced sensitivity to subtle changes of face features and their limited visual experience to faces caused by diminished social motivation. Last, our findings provide practical implications for designing robots and virtual characters for the intervention of children with ASD.

## Introduction

Robots and virtual reality are increasingly emerging in children’s daily lives. Robots teach children knowledge [1, 2] and accompany children as social partners [3]. Virtual reality provides children interesting and interactive virtual learning environments (see [4] for a review). In the clinical settings, robots and virtual reality have been used in the intervention for children with special needs, for instance, children with Autism Spectrum Disorder (ASD) (see [5] for a review). ASD is a neurodevelopmental disorder characterized by persistent deficits in social communication and social interaction, and restricted, repetitive patterns of behavior, interests or activities [6]. The core symptoms of ASD are the deficits in social interactions and communications (e.g., [7]). Leveraging on robots and virtual reality, researchers are striving to improve children with ASD’s social skills, including reciprocal interactions [8], gaze behaviors [9], gestures [10], and proximity regulation [11]. Thus, it is important to know what kinds of facial appearance of robots children would prefer, more human-like or machine-like. Tung [12] found that typical children prefer robots with a moderate level of anthropomorphic appearance to robots with highly human-like appearance among a wide range of robots, Woods [13] revealed that children rated human-machine robots as friendly and human-like robots as aggressive, and Peca and colleagues [14] found that both typically developing (TD) children and children with ASD like robots with cartoon-like appearance most among only six robots. To our best knowledge, only one study [14] has investigated whether children with ASD have specific preferences for the facial appearance of robots or virtual characters relative to TD children. Given children with ASD have reduced interests to social information [15], we would argue that this is not a trivial question since their preference would impact the efficacy of the interventions using virtual reality and robots.

The uncanny valley phenomenon provides an excellent behavioral window to answer this question. The term “uncanny valley” refers to people’s response to a human-like artefact would abruptly shift from high affinity to revulsion when the artefact approaches but fails to attain an actual human appearance [16]. This abrupt decrease in affinity is termed the uncanny valley. Although the existence of the uncanny valley is still inconclusive considering the conflicting existing evidence (e.g., [17–24]), it has been validated in adults with various types of stimuli, including stimuli ranging from mechanical robots to human-like robots [18, 21], from avatars to human [24], from mechanical-looking humanoids to human [20, 22], and from inanimate faces to human faces [19, 23].

It is still under debate when the uncanny valley effect emerges in young children. For instance, one study found the uncanny valley effect emerges at as early as 12 months old [25], while another study did not find the uncanny valley effect until 9 years old [26]. It is also crucial to examine whether children with ASD would display the uncanny valley effect since it would affect their preference for the virtual characters or robots in the intervention. Children with ASD have limited visual experiences of human faces due to their diminished social motivation [15], and they are less sensitive to featural and configural modifications of faces, such as the changes of eye sizes, the changes of distance between the eyes [27, 28]. Considering the important role of eye contact and mind perception in the uncanny valley, Schein and Gray [29] suggested that individuals with autism should be less likely to show the uncanny valley effect as they viewed the eyes less, though they have not provided experimental evidences. As the uncanny valley is critically dependent on the perceptual experience with real human faces [25] and the ability to detect the perceptual mismatch of the human-like artifacts [30], we also hypothesize that the uncanny valley effect is diminished or even absent among children with ASD.

To test our hypothesis, we used well-established stimuli, i.e., enlarged eye size, which has been repetitively shown effective in inducing the uncanny valley effect in studies of adults [23, 31]. To make our task age-appropriate and child-friendly, we used the two-alternative forced choice paradigm to measure their preference for different stimuli, as opposed to the more demanding scale ratings. To make sure children with ASD indeed look at the manipulated facial features, we also used an eye tracking device to monitor their gaze throughout the experiment.

## Materials and methods

### Participants

Twenty-six TD children (age range: 5.0–7.4 years, mean age: 6.30 years, SD = 0.75, one girl) and 26 children with ASD (age range: 4.9–7.6 years, mean age: 6.02 years, SD = 0.73, one girl) took part in this study. All children with ASD were diagnosed with ASD by professional clinicians according to the DSM-V [6]. We further confirmed the ASD diagnosis according to the Chinese version of Autism Spectrum Quotient: Children's Version (AQ-Child) [32] and the Social Responsiveness Scale (SRS) [33]. The two groups were matched on the chronological age, the verbal mental age (VMA, measured by Peabody Picture Vocabulary Test), and the non-verbal IQ scores (measured by Raven’s Test, see Table 1 for detailed information). Written informed consent was obtained from all children included in the study and their parents. All procedures performed in this study were approved by the Ethics Committee of School of Psychological and Cognitive Sciences, and were in accordance with the ethical standards of the 1964 Helsinki declaration and its later amendments or comparable ethical standards.

**Table 1:** Participant characteristics of TD group and ASD group.

		*N*	Male/ female	Mean age in years (SD)	NVIQ^a^ raw score	Standardized NVIQ (SD)	PPVT (SD)	VMA (SD)
ASD		26	25/1	6.30 (0.75)	32.15 (8.70)	105.65 (9.90)	101.00 (17.74)	7.27 (0.72)
TD		26	25/1	6.02 (0.73)	32.65 (8.09)	110.17 (9.37)	108.38 (19.96)	7.65 (1.09)
Difference (*t* test)^b^	ASD vs. TD	N/A	N/A	1.33	-0.21	-1.65	-1.41	-1.50

^a^ NVIQ was measured by the Combined Raven Test (CRT-C2).

^b^ all *Ps* > 0.1.

### Materials

**Fig 1:**
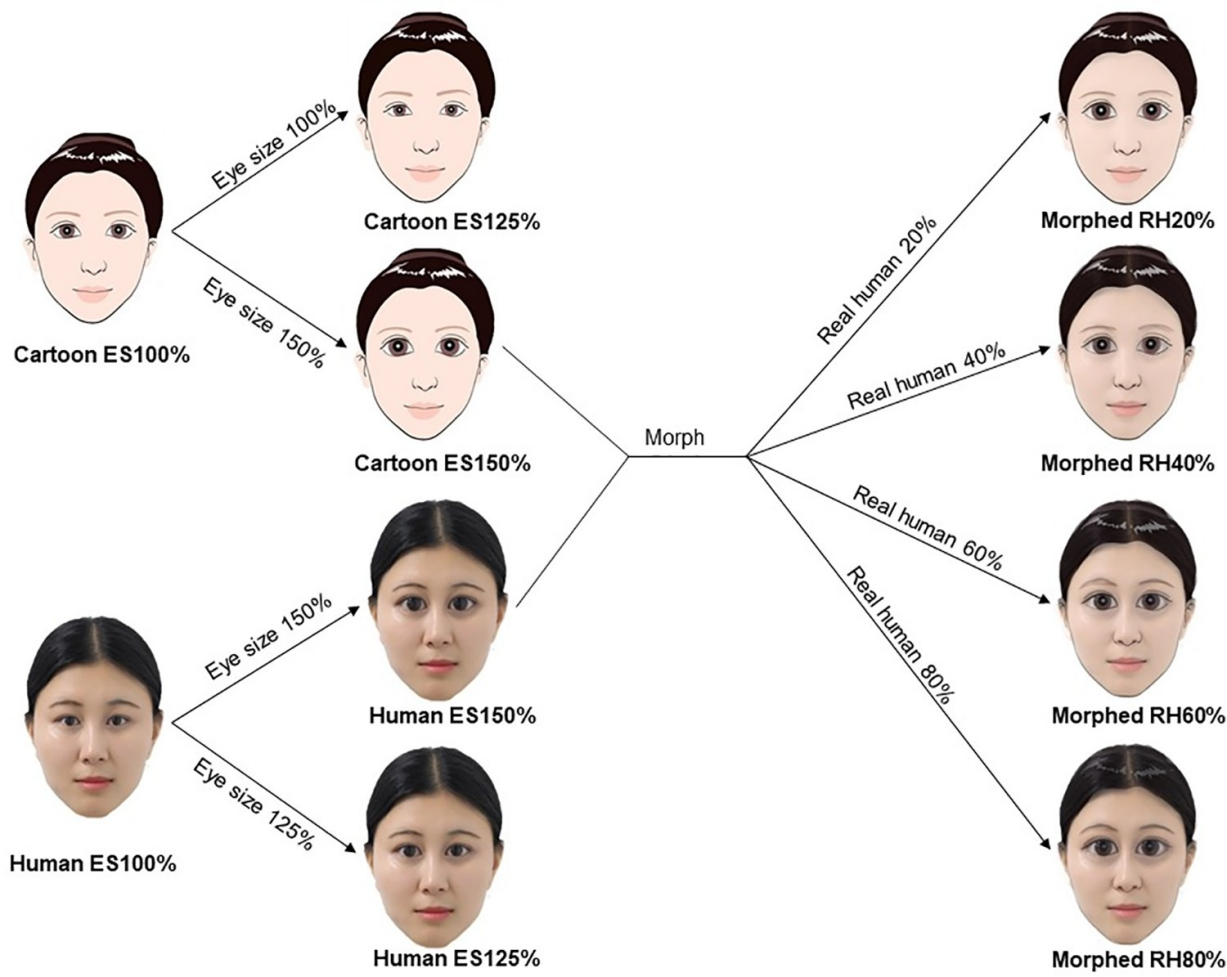
Materials used in this study and their relationship. The eye sizes (ES) of a cartoon face and a human face were first enlarged from 100% to 125%, and then to 150%. Thus, we had three cartoon images and three human images with eye sizes of 100%, 125%, and 150%, respectively. The cartoon face with 150% eye size was gradually morphed into the human face with 150% eye size, the percentage of real human (RH) were 20%, 40%, 60% and 80%. This resulted in four morphed images, as shown on the right column of the figure.

To create the experimental material, we used similar manipulations on face pictures as in one of the early studies [23]. We enlarged the eyes to different proportions, which have been shown to lead to the uncanny valley effect [23]. We also morphed the artificial faces with real human faces to generate images of different degrees of realism [34]. We used a computer graphics image of an adult female face (cartoon face) as the artificial face image and a 24-year old Chinese female face as the human face. We first scaled the eye size of these two images from the original size (100%) to the medium size (125%), then to the largest size (150%) using Adobe Photoshop CS6 (Fig 1). Thus, we obtained three cartoon images and three human images with eye sizes of 100%, 125%, and 150%, respectively. Then, we morphed the cartoon face image with 150% eye size into the human face image with 150% eye size using Abrosoft FantaMorph software. The morphing ratios—the percentage of real human image—were 20%, 40%, 60%, and 80%. Thus, we obtained ten images and normalized them to 500 × 700 pixels. In our process of morphing, we complied with the detailed guideline for morphing described in Cheetham and Jäncke [35]. The two endpoint images should be similar to each other to avoid artifacts [32], e.g., similar configural cues, similar ages, direct gaze, neutral facial expression. We chose a cartoon image rather than a robot image as one of the two endpoint images was simply because the cartoon face is more similar to the human face in terms of configural cues, ages, gaze direction, and facial expression. The individual in the images has given written informed consent to publish these images. We recruited 17 TD adults and did a plot study to test the validity of our stimuli for inducing the uncanny valley effect. This pilot study replicated the results of the Experiment 3 in Seyama and Nagayama [23].

### Apparatus and procedure

The participants were seated 60 cm from a display monitor with a screen resolution of 1024 × 768 pixels. Their eye movements were recorded by a Tobii X120 eye tracker (sample rate: 120 Hz). Participants were first engaged in a five-point calibration procedure. In the formal experiment, we presented each participant 90 pairs of images, which included all possible two-image combinations for the ten images and the order of image-pairs was randomized. The image pairs were presented at the center of the left and right halves of the screen and their left-right order were counterbalanced. The distance between the centers of the two images was 512 pixels. The participants were required to make a two-alternative forced choice to indicate the face they liked more by pressing the left or the right button on a mouse. Their eye movements were monitored. As mentioned above, we did not employ the five-point scale to measure children’s pleasantness for the images, because it is demanding for young children, especially for children with ASD, to evaluate to what degree they liked the images. The two-alternative forced choice is much easier for them. To make all participants understand our task, we showed them two kinds of snacks before the formal experiment and asked which one they liked more. One snack was that they liked, the other was that they disliked. All of them chose the one they liked more according to their parents’ reports. We told them to make similar choices as in snacks choosing, and to choose the one they like more between the two images displayed on the screen.

### Data analysis

We first calculated the number of times that a given image was selected as the preferred. This count was called preference index and calculated for each image and each individual participant. Theoretically, this index ranged between 0 and 9 with a larger number reflecting more preference.

Given that this frequency data did not meet the assumption of independent sampling, we used a modified version of the Bradley-Terry model to estimate the probability of choosing an image [36]. As shown by previous research [23] and our pilot data, the conditions that constituted the uncanny valley only included the ones with 150% eye size (Fig 1). Thus, our modified Bradley-Terry model only utilized the data obtained with the cartoon image with 150% eye size, the four morphed images and the human image with 150% eye size. We considered how a binary decision was made between two images:

$$Pr(i>j)=\alpha_{i}/(\alpha_{i}{+\alpha }_{j})$$ (1)

$$logit[Pr(i>j)]=log(\frac{Pr(i>j)}{1-Pr(i>j)})=\lambda_{i}-\lambda_{j}$$ (2)

where *i* and *j* are the index labels for the *i*th and *j*th image, respectively. α_i_ denotes participants’ preference for the *i*th image, Pr(*i*>*j*) denotes the probability that the *i*th image was selected over the *j*th image. With a logit function, e.q. 2 signifies that a binary decision is made by computing a relative preference, quantified as the natural log of the difference between the two probabilities.

Participants’ preference was influenced by covariates, including the degree of the realism of the image and the group a participant belonged to. We included these factors in the model and estimated the log probability of the *i*th image:

$$\lambda_{im}=\sum_{k=3,4,5,6,7,8}\gamma_{i}I(i=k)+\eta_{1}{{(x}_{i}-80\%)}^{2}+\eta_{2}{{(x}_{i}-80\%)}^{2}*I(m=1)$$ (3)

where *I()* is the indicator function, *k* is the index number of an image: *k* = 3 for the cartoon image with 150% eye size; *k* = 4 to 7 for the four morphed images with the degrees of realism increasing from 20% to 80%, respectively; *k* = 8 for the human image with 150% eye size. *x*_i_ represents the percentage of realism (i.e., 0%, 20%, 40%, 60%, 80% and 100%) of the *i*th image, *m* represents participants’ group label (TD group: *m* = 1; ASD group: *m* = 0). γ_i_ denotes the influence of the *i*th image. η_1_ denotes the relative change in preference as a function of deviation from 80% of realism where the uncanny valley dips the most. The coefficient η_2_ for the interaction term denotes the group effect of being a TD child. Note γ_i_, η_1_ and η_2_ are free parameters which are estimated from model fitting.

**Fig 2:**
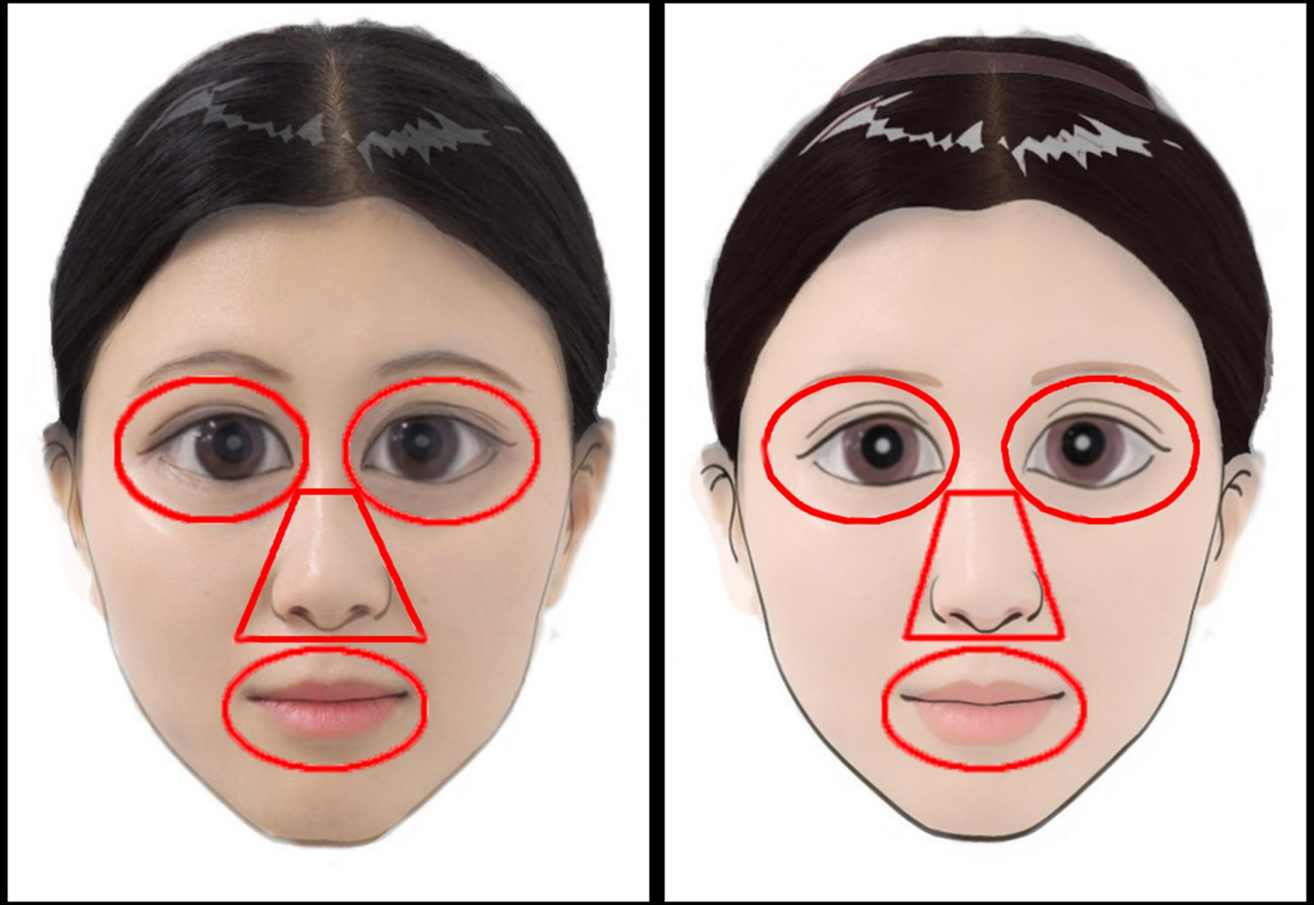
Samples of face stimuli used in the experiment. Areas of interest, including eyes, nose and mouth regions, are highlighted with red contours.

For eye tracking data, we extracted fixations from the raw gaze data according to the definition specified by Tobii (I-VT fixation filter) [37]. Specifically, minimum fixation duration was set at 100 ms within a velocity of 30 deg/s. We defined four areas of interests: the eyes area (the two eyes), the mouth area, the nose area and the face area (AOIs; Fig 2). We computed the proportions of fixation time on the eyes area, the mouth area and the nose area by dividing them into the total fixation time on the face area. These proportions of fixation time were averaged for each image and for each group. Besides AOI analysis, we created heat maps for fixation durations and compared them between groups by using *iMap* toolbox [38, 39].

## Results

### Image-specific preferences

**Fig 3:**
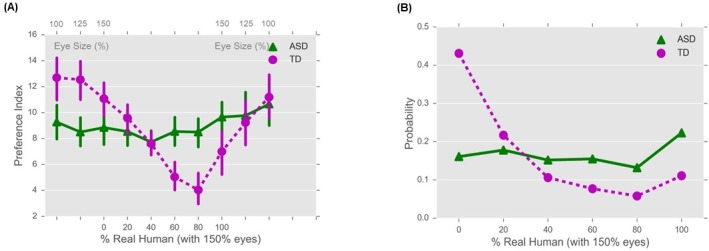
Preference for each image. (**A**). Preference indices plotted as a function of image. Upper X-axis: scaling factor for eye size, lower X-axis: degree of realism. Error bars are 95% confidence intervals. Data shown in the dash-line box are used for fitting the modified Bradley-Terry model. (**B**). Probability of choosing the five selected, morphed images with increasing degree of realism. X-axis: real human percentage, Y-axis: probability of choosing an image estimated from the modified Bradley-Terry model.

The TD group and the ASD group showed different preferences for the images tested, as revealed by the preference indices. Fig 3A showed that the TD group exhibited the uncanny valley effect: the preference indices decreased with the enlargement of the cartoon eye size, and further dropped with increasing percentage of realism in the cartoon face till the realism reached 80%. It then increased with decreasing eye size of the human face images. In contrast, this systematic pattern was absent for the ASD group—their preference indices remained similar across images.

Our modified Bradley-Terry model fitted the data reasonably well (Hosmer-Lemeshow goodness of fit test, *p* = 0.31). Its results indicated that realism in the morphed face image indeed made a difference with η_1_ = 1.318e-03, *p* < .0001. More importantly, we found significant group effect with η_2_ = 2.806e-04, *p* < .0001. Compared with children with ASD, TD children showed less preference for these images. We also computed participants’ probability of choosing each image based on fitted model parameters and Eq 1 (Fig 3B). Indeed, the TD group’s probability of choosing these images first decreased and then increased as the realism of these images increases, and exhibited a pattern of the uncanny valley. Whereas, the ASD group exhibited similar probability across images, and thus the uncanny valley was absent among them.

To examine the effect of enlarged eyes without face morphing, we selectively analyzed the three cartoon faces and the three human faces with different eye sizes. We conducted a 2 (image types: cartoon vs. human) × 3 (eye sizes: 100% vs. 125% vs. 150%) × 2 (groups: TD vs. ASD) repeated measures ANOVA on preference indices. Results revealed significant main effect of eye size, *F*(2, 100) = 12.70, *p* < .001, η_p_^2^ = .20, group, *F*(1, 50) = 14.55, *p* < .001, η_p_^2^ = .23, group × image type interaction, *F*(1, 50) = 5.01, *p* = .030, η_p_^2^ = .09, group × eye size interaction, *F*(2, 100) = 5.64, *p* = .005, η_p_^2^ = .10, and eye size × image type interaction, *F*(2, 100) = 3.13, *p* = .048, η_p_^2^ = .06. Simple main effect analyses of the group × image type interaction revealed that the TD group preferred the cartoon images to the human images, *F*(1, 50) = 5.19, *p* = .027, η_p_^2^ = .09, while the ASD group have similar preferences for the cartoon images and the human images, *F*(1, 50) = .79, *p* = .379, η_p_^2^ = .02. It also revealed that the TD group showed more preference for the cartoon images than the ASD group, *F*(1, 50) = 13.12, *p* = .001, η_p_^2^ = .21, while no group difference was found for the human images, *F*(1, 50) = 0.72, *p* = .400, η_p_^2^ = .01. Simple main effect analyses of the group × eye size interaction revealed that the TD group preferred images with different eye size differently, *F*(2, 49) = 13.25, *p* < .001, η_p_^2^ = .35, but the ASD group showed similar preferences for the images with different eye sizes, *p* = .088, η_p_^2^ = .10. More specifically, TD group preferred the face images with 100% eye size to the images with 125% eye size and preferred the images with 125% eye size to the images with 150% eye size (*p*s < .018, all *p* values were corrected by FDR). These results confirmed that children with ASD were indifferent to differences of images, including the eye size difference and the cartoon vs. human difference. On the other hand, TD children demonstrated systematical preferences: their preferences dropped with the increasing eye size and with the transition from cartoon to human faces. For images with different realism, TD group’s preference index for the image with the highest realism (i.e., the face with 80% realism) was the lowest; for the three human faces with different eye sizes, their preference index for the one with the largest eye size (i.e., human face with 150% eye size) was the lowest (Fig 3A).

### Fixation duration

**Fig 4:**
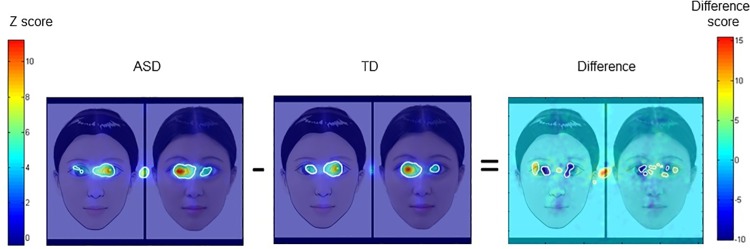
Results from *iMap* analysis of eye-tracking data. Two heat maps are separately shown for ASD group and TD group. Their difference constitutes a difference map, as shown in the right panel. The colors represent Z scores of fixation durations, with warm colors standing for longer fixation durations and cold colors for shorter fixation durations. Regions of significant difference are marked by white contours in the difference map (significance level set at alpha = 0.05, two-tailed).

We compared the proportional fixation durations averaged across ten images between the two groups for the eyes, the nose, and the mouth regions separately. Independent-sample *t*-tests revealed that two groups spent similar proportions of time looking at these AOIs (*p*s > .05, Cohen’s *d* < 0.51, all *p* values were corrected by FDR). We also conducted a 10 (image types) × 2 (groups: TD vs. ASD) repeated measures ANOVA on the proportional fixation durations on eyes, which was the region enlarged for most stimuli. No significant effect was found for the main effect of image type, the main effect of group, and the interaction between image type and group (*p*s > .05, η_p_^2^ < .03). Finally, we made heat maps and difference maps based on the fixation data using the *iMap* approach (Fig 4). As the difference map showed, compared to the TD group, the ASD group fixated less on the pupils, and more on the area between the two eyes, the region peripheral to the pupils, and the central fixation area (*p* < .05).

## Discussion

We found that TD children showed a pattern of the uncanny valley: with the realism increasing, TD children’s preference for face images first declined to a valley, then increased. This pattern was confirmed by our modified Bradley-Terry model: TD children’s probability of choosing an image first decreased and then increased with the realism of images increasing on the continuum. Moreover, TD children’s preference for the cartoon images and the human images declined with the enlargement of the eye size. However, the uncanny valley effect was absent in children with ASD: their preference remained largely the same as the realism increased, which was validated by the steady probability of choosing an image estimated by the modified Bradley-Terry model. In addition, the ASD group showed similar preferences for the images with different eye sizes, they also showed similar preferences for the cartoon images and the human images.

TD group’s preference for the image sequence in the present study showed the uncanny valley, which shared a similar pattern as the adult’s pleasantness in Seyama and Nagayama [23]. Therefore, the enlargement of the eyes could also cause the uncanny valley effect in TD children. Previously, some studies confirmed the existence of the uncanny valley [18, 20, 21, 22, 23], while some other studies failed to find the effect [17, 19, 24]. This inconsistency is not totally surprising since different studies manipulated the human-likeness quite differently [34], such as morphing a nonhuman face into a human face [17, 19], enlarging the eyes of the faces [23], manipulating the kinematic features of walking avatars [24], or selecting a number of stimuli rated with various human-likeness [21]. Our findings, along with others, support the uncanny valley for the specific manipulation used here.

Two theoretical explanations have been proposed to explain the uncanny valley. The category uncertainty theory asserts that the uncanny feeling is caused by an inability to determine an entity’s category when an entity transits from one category to another, e.g. from non-human to human [40–42]. The perceptual mismatch theory asserts that the uncanny feeling is caused by the mismatched human likeness of an entity’s features, e.g., a human face with mechanical eyes [29, 43, 44]. Recent empirical studies have found evidence that favors the perceptual mismatch theory as opposed to the category uncertainty theory. MacDorman and Chattopadhyay [30] found that the stimulus rated as the most ambiguous was neither the eeriest nor the coldest (also found in MacDorman & Chattopadhyay [45]), and that stimuli with features of inconsistent realism were rated as eerier and colder than stimuli with features of consistent realism (also found in Chattopadhyay & MacDorman [43]). Besides, Mathur and Reichling [21] also found that category confusion does not mediate participants’ ratings of likability. Our manipulation with enlarged eyes is one of the techniques that lead to perceptual mismatch [23, 31, 34]. Thus, the findings of the uncanny valley among TD children provide further supporting evidence for the perceptual mismatch theory and extend its scope to young children.

A previous study failed to find the uncanny valley effect for children as young as our TD group. Brink and colleagues [26] found that children did not show the uncanny feelings for the human-like robot until 9 years old. Note that our TD children were between 5 to 7 years old. The discrepancy in the findings could be explained by the different types of tasks and stimuli used in previous and present studies. Brink and colleagues [26] used only three stimuli, i.e., a human-like robot, a machine-like robot and a humanoid robot, and they employed 4-point scale to measure children’s feelings for the stimuli. As we argued before, the scale rating is a demanding task for young children, while our two-alternative forced choice task is much easier to complete. Furthermore, the difference in stimuli might also contribute to the different findings of the two studies.

More importantly, we found that children with ASD did not show the uncanny valley effect. This finding confirmed our hypothesis and the prediction of Schein and Gray [29]. The AOI fixation analysis revealed that the TD group and the ASD group showed similar looking patterns: they spent similar proportions of time looking at the eyes either when the eye movement data were averaged across the ten images or when they were analyzed independently for each image. The AOI results indicated that the absence of the uncanny valley in children with ASD was not caused by their inattention to the manipulated features. The AOI finding also excludes the possibility that children with ASD may make a two-alternative forced choice without scanning the eyes area which conveyed the perceptual mismatch information in the present study [23, 34]. Two possible reasons could explain why the uncanny valley effect was absent in children with ASD: one is that they could not detect the perceptual mismatch features (i.e., the enlarged eyes on faces) and the changes of the realism on the faces, the other is that they were indifferent to these features or changes even though they detected them. Previous studies also found that individuals with ASD are unable to detect the subtle differences in eye sizes or in the distance between the eyes (e.g., [27, 28]). People typically can recognize subtle changes in a face and even label it as another face, but this ability is severely reduced among individuals with ASD (e.g., [26, 27]). The absence of the uncanny valley in children with ASD could also be explained by their limited visual experiences due to the diminished social motivation to the social stimuli and human faces [15]. Compared with TD children, they showed diminished social orienting: they looked less at people and faces and more at background [46, 47]; they viewed social motion more and non-social motion less [48, 49].

Infants did not show the uncanny valley effect until 12 months old implied that the early visual experience for faces played a central role in forming the face prototype, which contributed to the emergence of the uncanny valley effect [25]. As people with ASD have deficits in forming the prototype of faces [50, 51], we could speculate that the reduced visual experience of people with ASD leads to their deficits in forming prototype faces, which further causes their absence of the uncanny valley effect. But this needs further investigation.

Our AOI results that the TD and the ASD groups spent similar proportions of time looking at the eyes seem contradicted with the results of previous studies which demonstrated that children with ASD paid less attention to the eyes [52–55]. However, by examining these previous studies carefully, we found that it was stimuli type that caused the inconsistency in results: our stimuli were static images, while the stimuli of these previous studies were videos involved interpersonal interactions [52–55]. Empirical studies also exhibited that the types of stimulus affected participants’ social attention allocation [56]. Speer and colleagues [57] used four types of stimuli to track the gaze behaviors of children with and without autism, and found that children with autism spent significantly less time viewing the eyes than TD children only for the social dynamic stimuli. Chevallier and colleagues [56] also used three types of stimuli to measure the social attention of children with and without ASD, and found that children with ASD spent less time looking at social stimuli than TD children only for stimuli involved interaction. Previous studies which used static images as the stimuli confirmed our results, as they also found that the TD group and the ASD group spent similar proportions of time looking at the eyes (e.g., [58–60]). Therefore, though the two groups in the present study spent similar proportions of time looking at the eyes, they may still manifest diminished looking time on eyes when the stimuli are videos involved interaction or when they engage in social interaction in daily life.

The *iMap* analysis found that children with ASD fixated less on the pupils and more on the area between the two eyes and the region peripheral to the pupils, which is consistent with the previous findings [59]. Such a face scanning pattern is believed to help alleviate discomfort elicited by direct eye contacts in individuals with ASD (e.g., [61]). Though children with ASD fixated less on the pupils and more on the area between the two eyes and the region peripheral to the pupils, their attention was on the eyes. The finding that the two groups spent similar proportions of time viewing the eyes also supported this point. Thus, we tend to conclude that children with ASD attended to the eyes similarly as TD children but they could not detect perceptual mismatch features, or they simply did not have preference for these perceptual mismatch features. It led to the absence of the uncanny valley effect in children with ASD.

Our findings provide some implications for the design principles of robots and characters in virtual reality environments. The uncanny valley effect in TD children reflects their sensitivity to face features induced perceptual mismatch. TD children also showed more preference for cartoon faces than human faces. Thus, when designing characters to teach or to play with TD children, we should not only avoid features that induce perceptual mismatch, but also employ the cartoon faces to facilitate their interaction with the characters. As the uncanny valley was absent in children with ASD and they showed no preference for cartoon faces and human faces, we can put effectiveness of the intervention at the first place when designing the characters for the intervention of children with ASD. More cartoon-like characters might exaggerate social cues via the physical appearance [5] and lessen children with ASD’s anxiety in the interaction, and can promote the social skill learning. However, more human-like characters can facilitate the generalization of the learned social skills into daily life [5]. Therefore, it depends on the aim of the intervention when designing the characters for children with ASD’s intervention.

## Conclusions

Robots and virtual reality are increasingly employed in the intervention of the social deficits of children with ASD. However, these children’s attitudes towards the non-human characters deserve scrutiny given that their attitudes and preferences would affect their social behavior during intervention. Our study found that TD children, but not children with ASD, exhibited the uncanny valley effect when the artificial faces with mismatched facial features induced decreasing affinity. Particularly, TD children’s preference for the cartoon images and the human images declined with the enlargement of the eye size and increasing realism of face images. These effects were absent among children with ASD: they were rather indifferent to our manipulation of facial features. These findings provide evidence for the existence of the uncanny valley in younger children and favoring the perceptual mismatch theory that aims to explain the uncanny valley effect. The absence of the uncanny valley in children with ASD was possibly due to their reduced sensitivity to subtle changes of face features and their limited visual experience to faces caused by diminished social motivation. Our findings suggest that the virtual characters or robots should avoid inducing the uncanny effect for TD children, while their design should simply focus on the efficacy of intervention for children with ASD.
